# MAIT cells: Conserved watchers on the wall

**DOI:** 10.1084/jem.20232298

**Published:** 2024-10-24

**Authors:** Lilou Germain, Pablo Veloso, Olivier Lantz, François Legoux

**Affiliations:** 1 INSERM ERL1305, CNRS UMR6290, Institut de Génétique and Développement de Rennes, Université de Rennes, Rennes, France; 2 Institut Curie, PSL University, Inserm U932, Immunity and Cancer, Paris, France; 3 Laboratoire d’immunologie Clinique, Institut Curie, Paris, France; 4 Centre d’investigation Clinique en Biothérapie Gustave-Roussy Institut Curie (CIC-BT1428), Paris, France

## Abstract

MAIT cells are innate-like T cells residing in barrier tissues such as the lung, skin, and intestine. Both the semi-invariant T cell receptor of MAIT cells and the restricting element MR1 are deeply conserved across mammals, indicating non-redundant functions linked to antigenic specificity. MAIT cells across species concomitantly express cytotoxicity and tissue-repair genes, suggesting versatile functions. Accordingly, MAIT cells contribute to antibacterial responses as well as to the repair of damaged barrier tissues. MAIT cells recognize riboflavin biosynthetic pathway-derived metabolites, which rapidly cross epithelial barriers to be presented by antigen-presenting cells. Changes in gut ecology during intestinal inflammation drive the expansion of strong riboflavin and MAIT ligand producers. Thus, MAIT cells may enable real-time surveillance of microbiota dysbiosis across intact epithelia and provide rapid and context-dependent responses. Here, we discuss recent findings regarding the origin and regulation of MAIT ligands and the role of MAIT cells in barrier tissues. We speculate on the potential reasons for MAIT cell conservation during evolution.

## Teaser

Our understanding of MAIT cell biology is increasing rapidly. Here, Germain et al. discuss recent findings regarding MAIT cell functions in barrier tissues, with a focus on the role of pathogen- and microbiota-derived MAIT cell ligands.

## Introduction

Mucosal-associated invariant T (MAIT) cells are innate-like T cells specific for microbial riboflavin pathway–derived metabolites presented by the MHC class Ib molecule MR1. MAIT cells express a quasi-invariant TCR alpha (iTCRα) chain composed of the TRAV1 segment paired with TRAJ33 in mice and with TRAJ33, TRAJ12, or TRAJ20 in humans, and with a conserved CDR3α length (Reantragoon et al., 2012; Tilloy et al., 1999). Both MR1 and the MAIT iTCRα are under purifying selection (Boudinot et al., 2016), indicating evolutionary pressure to retain this antigenic specificity in the T cell repertoire. Yet, the role of the MAIT antigenic specificity in health and disease remains elusive. Herein, we will review the recent literature on the origin and distribution of MAIT ligands in vivo. The canonical MAIT ligands are metabolic adducts of the riboflavin biosynthesis pathway (Corbett et al., 2014) produced by pathogenic bacteria, but also by members of the intestinal microbiota. Intestinal dysbiosis induced by gut inflammation can increase the synthesis of MAIT ligands, resulting in MAIT cell activation (El Morr et al., 2024). The agonist microbial ligands for MAIT cells have the property to cross epithelial barriers and circulate in the body (El Morr et al., 2024; Legoux et al., 2019a), raising several questions regarding the regulation of the riboflavin biosynthetic pathway activity in the microbiota and of MAIT cell responses to TCR stimulation in distant sites.

MAIT cells can kill bacteria-infected cells (Le Bourhis et al., 2010, 2013) and contribute to the clearance of bacterial pathogens in a TCR-dependent manner in vivo (Wang et al., 2018; Zhao et al., 2021). In addition, MAIT cell TCR triggering drives the expression of tissue-repair genes (Hinks et al., 2019; Leng et al., 2019), and MAIT cells accelerate skin wound healing and intestinal barrier repair in vivo (Constantinides et al., 2019; du Halgouet et al., 2023; El Morr et al., 2024; Varelias et al., 2018). MAIT cells across mammalian species acquire an evolutionarily conserved transcriptional program marked by the co-expression of tissue repair and cytotoxicity genes (Bugaut et al., 2024), further emphasizing the importance of these two functions in MAIT cells. In this review, we will discuss the implication of these findings and speculate about the potential reasons for MAIT cell conservation (or loss) during evolution.

## Origin and evolution of MAIT cells

Since the discovery of MAIT cells (Tilloy et al., 1999; Treiner et al., 2003), the striking conservation among mammalian species of both the MAIT iTCRα genes and the MR1 molecule indicated that the antigen specificity of MAIT cells is crucial for MAIT cell function(s) under selective pressure (Boudinot et al., 2016; Huang et al., 2009). MR1 appeared in the ancestors of mammals as no related molecule is found in reptiles, fish, and amphibians (Boudinot et al., 2016). MR1 is the most conserved MHC molecule in mammals as the α1 and α2 domains of human MR1 are 86% identical with murine MR1 and 70% with opossum MR1 (Boudinot et al., 2016). Contrary to classical MHC molecules, MR1 is under strong purifying selection with an excess of synonymous nucleotide substitutions in the α1 and α2 domains, suggesting a selective pressure and an important role in maintaining antigen specificity (Huang et al., 2009).

The most potent ligands for MAIT cells are produced by the non-enzymatic condensation of the riboflavin biosynthesis pathway intermediate 5-A-RU with glyoxal or methylglyoxal to form 5-(2-oxoethylideneamino)-6-D-ribitylaminouracil (5-OE-RU) and 5-(2-oxopropylideneamino)-6-D-ribitylaminouracil (5-OP-RU), respectively (Corbett et al., 2014). As 5-A-RU is not synthesized by animal cells, only microbes can provide 5-A-RU, but glyoxal and methylglyoxal can originate from various metabolic pathways present in both microbes and host cells.

Consistent with the phylogenetic conservation of both TRAV1 and MR1, murine MAIT cells recognize human MR1 loaded with 5-OP-RU and vice-versa as well as cattle MR1 (Huang et al., 2009; Le Bourhis et al., 2010; López-Sagaseta et al., 2013). More recently, interspecies cross reactivity of the MAIT TCR toward MR1:5-OP-RU from various species was extended to sheep and pig MR1 although the number of MAIT cells in pigs was extremely low despite a perfectly functional pig MR1:5-OP-RU tetramer recognizing MAIT cells from other species (Edmans et al., 2024). Bats harbor a high number of MAIT cells that are cross reactive toward cattle MR1:5-OP-RU (Leeansyah et al., 2020). The intensity of staining by the MR1:5-OP-RU tetramer varies according to the species of both MR1 and MAIT cells partly in relation to the variable proportion of MAIT cells expressing CD8 (Edmans et al., 2024; Souter et al., 2022), which can increase binding affinity to MR1.

Consistent with interspecies cross-reactivity, TRAV1 and TRAJ33 are among the most conserved TRAV and TRAJ genes in mammals (Boudinot et al., 2016). The TRAV1 gene is a one-member family located at the 5′ end of the TRAV locus in all sequenced species. The conservation of TRAV1 is remarkable given the plasticity of the TRAV locus, which exhibits very variable numbers of TRAV genes from one species to another (45 in humans to >350 in cattle) and even duplications/deletions of whole parts of the locus in some mouse strains. In contrast with the TRAV locus, the MR1 region is very well conserved with syntenic regions between species (Boudinot et al., 2016). Surprisingly, the TRAV1 locus has been lost in three groups of mammals (lagomorphs, armadillo, and carnivorous) and the MR1 gene is either pseudogenized or lost in the exact same species (Boudinot et al., 2016). Because of the plasticity of the TRAV locus, this striking co-evolution suggests that when the TRAV1 was lost in some species, the selective pressure on MR1 was relaxed leading to its pseudogenization or even loss. Thus, the only selective evolutionary pressure on MR1 is the education of and presentation of antigens to TRAV1^+^ cells (Mondot et al., 2016).

These data indicate that the function of MAIT cells under evolutionary pressure is associated with antigen specificity. Although most (>85%) bacterial species have the genes necessary for 5-OP-RU generation (Mondot et al., 2016), only a few of them express this pathway at steady state and during diseases (El Morr et al., 2024). MAIT cells could be involved in the regulation of gut homeostasis by sensing the loss of physiological gut hypoxia leading to higher 5-OP-RU production by the gut microbiota (see Origin and dynamics of MAIT agonist ligands section), or in the response against pathogenic bacteria. In the latter hypothesis, the bacteria should be intracellular or growing slowly to account for the small number and the slow proliferation of MAIT cells as compared with bacteria. Ubiquitous slow-growth pathogens such as *Mycobacteria* may be relevant as suggested by the epidemiological link between a polymorphism potentially modifying MR1 expression and the incidence of severe tuberculosis in a Vietnamese cohort (Mondot et al., 2016; Seshadri et al., 2017). In any case, the pathogen(s) responsible for this evolutionary pressure may not be present anymore in the very clean environment of current Western societies.

Regulating gut homeostasis by sensing changes in bacterial metabolism or fighting pathogenic bacteria may require distinct functionalities: secretion of IL17A, IL-22, Amphiregulin, or other tissue-repair mediators in the first case or secreting granulyzine, IL4I (generating H2O2), IL-17F, or IFN-γ, for instance, to fight invasive bacteria (Garner et al., 2023; Salou et al., 2018). Both functions can be exerted by MAIT cells according to the context of stimulation as the evolutionarily conserved functional program they express is a mixed type 1 and 17 program. In most species, this mixed type 1/17 program is already acquired in the thymus, while in rodents, distinct MAIT1 and MAIT17 are found in the thymus (Bugaut et al., 2024; Koay et al., 2019; Legoux et al., 2019b). However, a mixed 1/17 effector program is acquired in the periphery in rodents, at steady state in the intestine (Bugaut et al., 2024) or after bacterial infection in the lung (Chen et al., 2017; Wang et al., 2018). The MAIT17 program is associated with migration into the parenchyma while the repair function is thought to be triggered by TCR signaling (du Halgouet et al., 2023; Hinks et al., 2019; Lamichhane et al., 2019; Leng et al., 2019). Altogether, these features of MAIT cells may explain the evolutionary importance of sensing 5-OP-RU in several physio-pathological settings in the gut and the lung.

Notably, the evolutionary conservation, the developmental pathway, and the functionalities of MAIT cells are shared with another innate-like T cell subset: invariant natural killer T (iNKT) cells that recognize glycolipids presented by CD1d also using an iTCRα chain (Bendelac et al., 2007; Legoux et al., 2017). During bacterial infection, the ligands activating iNKT cells can be either exogenous provided by the bacteria or endogenous upregulated through a TLR-dependent mechanism by the bacteria (Mattner et al., 2005). In mice, contrary to MAIT cells, iNKT cells are mostly type 1 and preferentially located in the vasculature of the liver where they are very abundant (Geissmann et al., 2005). While MAIT cells are abundant in humans, they are very few in mice while it is the opposite for iNKT cells. In mice, once split according to type 1 or 17, iNKT and MAIT cell subsets are extremely similar regarding functions and tissue location. MAIT and iNKT cell numbers are positively correlated in the various organs (Salou et al., 2018) suggesting an absence of competition for peripheral niches. Yet CD1d^−/−^ mice, which selectively lack iNKT cells, exhibit increased MAIT cell frequencies (Koay et al., 2016). The opposite is not true as MR1^−/−^ mice have normal iNKT cell frequencies (El Morr et al., 2024). Thus, whether MAIT and iNKT cells compete for peripheral niches is unclear. Like MAIT cells, the few iNKT cells found in humans display a mixed 1/17 functional program (Bugaut et al., 2024). Since both innate-like T cell subsets share the same transcriptome programs, functional redundancy is likely when the effector modules are triggered by non-cognate signals such as IL-12, IL-18, IL-23, or IFN-α.

This leads to several questions:

What could be potential drivers for the selection of the MR1-MAIT system? The *MR1* gene was not found in the genome of the egg-laying mammal platypus (Boudinot et al., 2016), suggesting that the selection of *MR1* occurred after the separation of placental and marsupial mammals from monotremes about 200 million years ago (MYA) (Álvarez-Carretero et al., 2022). Thus, the selection of MR1 and MAIT cells may be associated with the loss of oviparity and the emergence of a genital tract separated from the digestive tract in marsupial and placental mammals. MAIT cells are present in the human female genital tract where they exhibit a distinct IL-17/IL-22 profile and respond to bacterial stimulation (Bister et al., 2021; Gibbs et al., 2017), yet their role in this tissue remains unclear.
Why were MAIT cells lost in carnivora? One possibility would be that the specific diet of carnivora induces intestinal hyperoxia because of a gut with limited adaptation to bacterial fermentation (Verbrugghe et al., 2012). The higher oxygen concentration in the intestinal lumen may select bacterial species producing high amounts of riboflavin and thus of 5-OP-RU. The resulting hyperactivation of MAIT cells would be deleterious contra-selecting this subset.
For the functions triggered by non-cognate stimuli, what are the respective roles of MAIT and iNKT cells in the different physio-pathological contexts? Assuming that MAIT and iNKT cells fulfill important evolutionarily conserved functions, is there another T cell subset displaying similar effector modules in lagomorphs that lack both MAIT and iNKT cells? This hypothesis is supported by the existence of another MHC class I molecule sharing some common evolutionary features with MR1 (Boudinot et al., 2016).

## Origin and dynamics of MAIT agonist ligands

The half-life of 5-OE-RU and 5-OP-RU in physiological conditions is relatively short (about 90 min) due to cyclization reactions in aqueous buffers (Mak et al., 2017). However, 5-OE/P-RU are stabilized by the formation of a reversible Schiff base with lysin 43 of MR1, a reaction that occurs in the endoplasmic reticulum (McWilliam et al., 2016). Neutralization of the positive charge of Lys43 induces a conformational change in MR1 leading to the export of the complex to the cell surface, enabling recognition by the MAIT TCR.

The riboflavin biosynthesis pathway gene *ribD*, which controls the synthesis of 5-A-RU, is strictly required for MAIT cell activation by both Gram^+^ and Gram^−^ bacteria (Corbett et al., 2014; Soudais et al., 2015). The rib pathway is widely distributed in bacteria, probably through horizontal gene transfers (Abbas and Sibirny, 2011). Several species from diverse genera are unable to synthesize riboflavin (Mondot et al., 2016). Whether the lack of riboflavin biosynthesis pathway represents an evasion mechanism allowing pathogens to escape host detection is unclear. Notably, non-pathogenic bacteria beneficial to human health, such as lactic acid bacteria, also lack a riboflavin biosynthesis pathway. On the other hand, the riboflavin pathway is an essential virulence trait for numerous bacterial pathogens. In *Helicobacter pylori*, the first enzyme involved in riboflavin biosynthesis, the GTP cyclohydrolase II, confers a hemolytic phenotype to the bacterium (Bereswill et al., 1998). Similarly, in *Candida albicans*, the early steps of riboflavin biosynthesis (reductase and deaminase) are required for virulence (Becker et al., 2010).

A comparison of MAIT ligand synthesis across 47 bacterial strains grown in vitro confirmed that the riboflavin biosynthesis pathway is required to activate MAIT cells (Tastan et al., 2018). However, harboring the rib pathway is not sufficient, probably because the activity of the rib pathway is tightly controlled, at least in some species (Tastan et al., 2018). In particular, in some species, the riboflavin product flavin mononucleotide binds to and inhibits the transcription and/or translation of rib genes, a feedback control mechanism referred to as a “riboswitch” (Gelfand et al., 1999). Riboflavin and MAIT ligand synthesis are reduced when riboswitch-equipped bacterial species are cultured in the presence of riboflavin (Corbett et al., 2014). The amounts of riboflavin and MAIT ligands produced in the bacterial culture medium are positively correlated (Tastan et al., 2018). Although there are many exceptions, species from the Bacteroidetes and Proteobacteria phyla can produce high levels of riboflavin and MAIT ligands. By contrast, species from the Firmicutes and Actinobacteria phyla often lack a complete rib pathway (Magnúsdóttir et al., 2015), and produce limited amounts of ligands in vitro when they possess a full rib pathway (Tastan et al., 2018). It is unclear whether 5-OE/P-RU is actively secreted or passively released by bacteria in the environment. Bacterial metabolism can influence the activity of the rib pathway and the production of MAIT ligands. *Escherichia coli* produces more MAIT ligands under anaerobic conditions as well as on glucose and ribose carbon sources as compared with aerobic conditions and glycerol, lactate, or acetate carbon sources (Schmaler et al., 2018). Transcription of the rib operon is strongly and transiently induced in *Clostridioides difficile* upon oxidative stress (Neumann-Schaal et al., 2018), consistent with the role of riboflavin in resistance to oxidative stress (Ashoori and Saedisomeolia, 2014). Thus, species releasing low levels of MAIT ligands in a given culture medium may become strong producers under specific microenvironmental conditions (Tastan et al., 2018).

In the complex ecosystem of the colon, transcription of the rib pathway is stronger in the mucus layer as compared with the lumen, and this is associated with higher MAIT ligand concentrations in mucosal scrapes as compared with luminal contents (El Morr et al., 2024). The riboflavin pathway transcriptional activity is undetectable in colonic archea or fungi, and depletion of fungi does not reduce MAIT ligand concentrations in the gut (El Morr et al., 2024). Although most bacterial species living in the colon harbor a full rib pathway, transcription of the *ribD* gene is dominated by a few families belonging to the minor phyla Proteobacteria and Deferribacteres (El Morr et al., 2024). In particular, Desulfovibrionaceae contribute the most to *ribD* transcription in the lumen, while *Mucispirillum schaedleri* express the most *ribD* found in the mucus. Interestingly, these bacteria possess noncanonical metabolisms based on taurine respiration (in the case of *Bilophila wadsworthia*), sulfur reduction (*Desulfovibrio*), and nitrate reduction (*M. schaedleri*) (Loy et al., 2017). Both families resist oxidative stress owing to the expression of catalase and other oxygen-scavenging systems. The robust expression of a rib pathway may contribute to oxidative stress resistance. Indeed, riboflavin is used by some anaerobic bacteria to reduce oxygen and grow in contact with air (Khan et al., 2012; Seeley and Del Rio-Estrada, 1951). Thus, microbiota production of MAIT ligands may reflect bacterial needs for riboflavin to resist oxidative stress.

Dysanaerobiosis (the presence of oxygen in the gut lumen) is a hallmark of intestinal inflammation and is associated with an expansion of bacterial families able to survive oxidative stress or even to use oxygen as an electron acceptor for energy production through respiration. Dysanaerobiosis can be induced by streptomycin, which depletes bacteria synthesizing butyrate. The lack of butyrate, in turn, leads to reduced oxygen consumption by colonocytes and thus increased oxygen levels in the lumen (Rivera-Chávez et al., 2016). MAIT ligands are more abundant in the cecum of streptomycin-treated mice (El Morr et al., 2024). Administration of butyrate to streptomycin-treated mice reduces oxygen levels and MAIT ligand concentrations in the gut (El Morr et al., 2024), consistent with a link between oxygen and MAIT ligand levels in the gut. Interestingly, intestinal bacterial strains with evidence of co-evolution with their human hosts are more likely to be intolerant to oxygen as compared with bacterial strains with no such evidence of co-evolution (Suzuki et al., 2022). If aerotolerance is associated with strong riboflavin biosynthesis, then co-evolution with human hosts may result in reduced riboflavin production. In support of this hypothesis, bacterial species within the Firmicutes phylum show the strongest co-evolution with human hosts (Suzuki et al., 2022) and are generally poor producers of riboflavin and MAIT ligands (Tastan et al., 2018), while Bacteroidetes show the weakest co-evolution with human hosts and are strong producers of riboflavin and MAIT ligands (Tastan et al., 2018). Thus, we speculate that MAIT cells may primarily become activated in the presence of bacterial strains with a recent history of co-evolution with their hosts.

The dextran sodium sulfate (DSS) model of intestinal inflammation is also associated with dysanaerobiosis (Lee et al., 2022). DSS treatment leads to increased bacterial expression of the riboflavin pathway and strong production of both riboflavin and MAIT ligands in the cecum (El Morr et al., 2024). The increased *ribD* expression is largely attributable to *M. schaedleri* alone, which blooms during colitis, possibly owing to its resistance to oxidative stress (Loy et al., 2017). Thus, in this system, increased synthesis of MAIT ligands appears attributable to the expansion of a few strong producers rather than to a global increase in rib pathway activity across multiple bacterial phyla. Of note, the riboflavin biosynthetic pathway is also overexpressed in the *Helicobacter hepaticus* model of colitis (El Morr et al., 2024), suggesting a broad and generalizable response of the gut microbiota to inflammation.

In some patients with Crohn’s disease, intestinal dysbiosis is associated with an expansion of bacterial species capable of riboflavin biosynthesis, along with glutathione transport and cysteine biosynthesis, consistent with increased redox tolerance (Morgan et al., 2012). Similarly, an increased abundance of riboflavin-competent bacteria is reported in patients with colorectal cancer (Feng et al., 2015) and rheumatoid arthritis (Thompson et al., 2023). Whether this translates into stronger synthesis of MAIT ligands is unknown.

The production of MAIT ligands in the gut lumen can potentially affect MAIT cell biology in distant sites because 5-OP-RU rapidly crosses epithelial barriers as 5-OP-RU painted on the skin or administered by gavage is found in the thymus within 30 min (Legoux et al., 2019a). 5-OP-RU can cross the intact gut wall (including epithelium, lamina propria, and muscularis) within 10 min ex vivo (El Morr et al., 2024). Given the hydrophilic nature of 5-OP-RU, there may be a transporter mediating epithelial passage. The rapidity of the passage suggests that MAIT cells may respond almost immediately to ligands produced by the microbiota such as during dysbiosis, even as the epithelial barrier remains intact. This contrasts with conventional T cell responses to classical peptidic antigens, which require sampling by dendritic cells across the intestinal epithelium prior to processing, migration, and presentation in neighboring lymph nodes. Since MR1:5-OE/P-RU complexes are rapidly internalized and degraded by antigen-presenting cells (McWilliam et al., 2016), MR1 presents a picture of the current metabolic state of the microbiota. Thus, MAIT cells may monitor a metabolic pathway indicative of oxidative stress and intestinal inflammation, almost in real-time and across the gut barrier.

In addition to 5-OE/P-RU, MR1 can present other molecules to MAIT cells, but with lower affinity than 5-OE/P-RU. In particular, the bile acid metabolite cholic acid 7-sulfate (CA7S) can form either by sulfate conjugation of the host molecule cholic acid by host sulfotransferases or by deconjugation of the bile acid metabolite tauro-CA7S by microbial bile salt hydrolases (Ito et al., 2024). Germ-free mice lack CA7S, pointing to tauro-CA7S as a major source of CA7S in vivo. MR1 binding affinity to CA7S is 10^3^- to 10^6^-fold lower than to 5-OP-RU, possibly due to the lack of Schiff base formation with MR1 (Ito et al., 2024). However, as discussed in MAIT cell ontogeny section, CA7S is present at high concentrations in vivo, which can compensate its low affinity for MR1. Additional MAIT ligands include the riboflavin intermediate 6,7-dimethyl-8-D-ribityllumazine (RL-6,7-diMe), which has a 10^3^-fold lower affinity for MR1 as compared with 5-OP-RU (Ito et al., 2024). The role of MAIT ligands in MAIT cell ontogeny will be discussed in the next section.

## MAIT cell ontogeny

**Figure 1.**
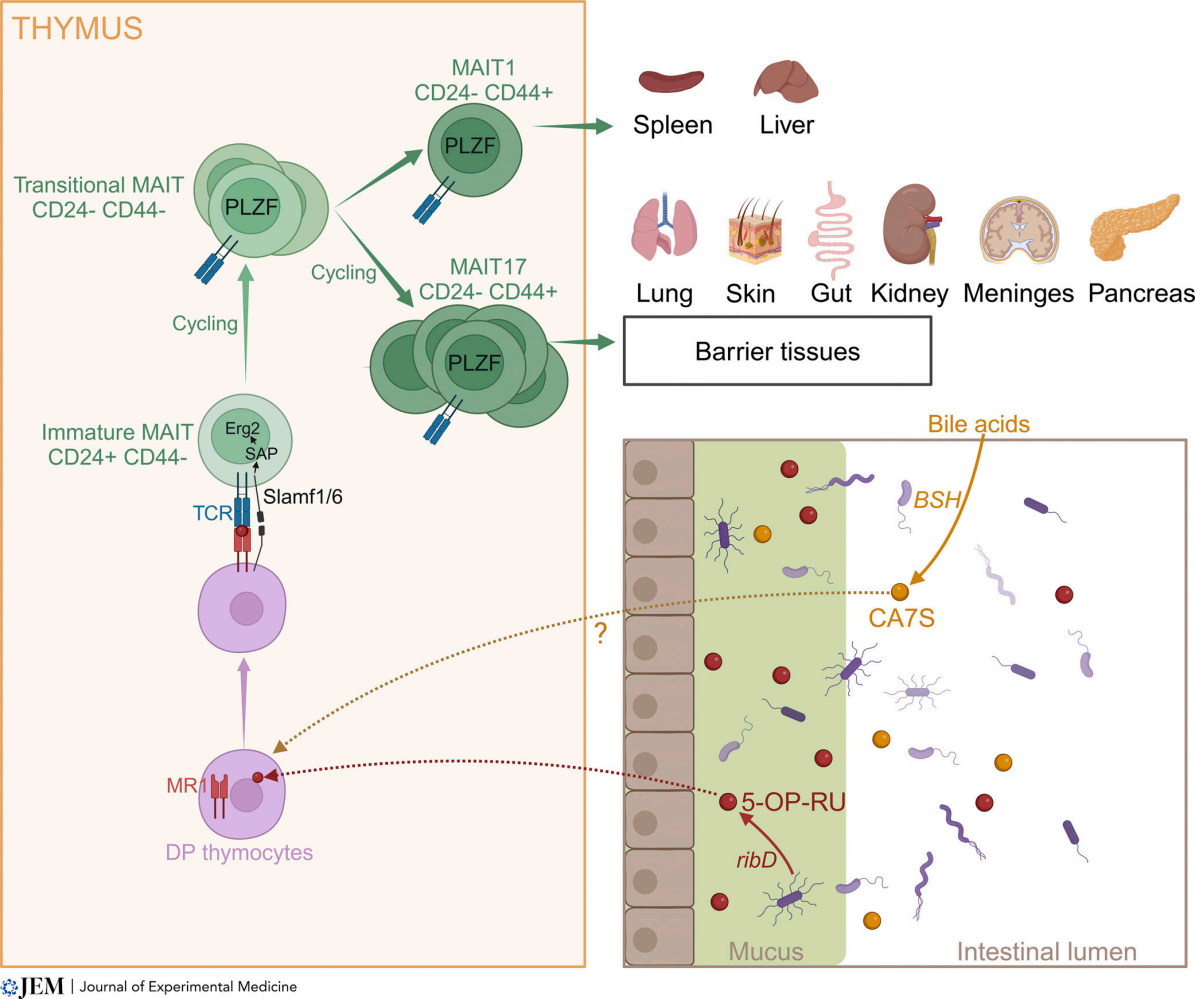
**MAIT cell development in mice.** 5-A-RU-derived MAIT ligands, mostly produced in the colon mucosa, travel to the thymus and contribute to the positive selection and/or intrathymic expansion of MAIT cells, in particular the MAIT17 subset. Upon positive selection by CD4^+^CD8^+^ DP thymocytes, MAIT cell precursors express PLZF and differentiate into either MAIT1 or MAIT17 cells. Expression of distinct homing proteins in each subset targets MAIT1 cells preferentially to the spleen and liver, while MAIT17 cells are home to barrier tissues such as lung, skin, and gut lamina propria. In addition to 5-A-RU-derived ligands, a bile acid metabolite primarily produced by bacterial bile salt hydrolases (BSH) can also be found in the thymus and modulate MAIT cell development. Created with Biorender.

Like conventional T cells, MAIT cells develop from CD4^+^CD8^+^ double positive (DP) immature thymocytes. However, while conventional T cells undergo positive selection by classical MHC molecules at the surface of epithelial cells, MAIT cell precursors engage with MR1 presented by other hematopoietic cells, in particular DP thymocytes (Legoux et al., 2019b; Seach et al., 2013) (Fig. 1). Positive selection by hematopoietic cells instead of epithelial cells induces expression of the master transcription factor promyelocytic leukemia zinc finger (PLZF) in MAIT cells (Legoux et al., 2019b). PLZF drives the acquisition of an effector, tissue-resident phenotype in T cells and as such represents a lineage-defining transcription factor for innate-like T cells (Thomas et al., 2011). Transgenic expression of MHC class II on thymocytes leads to the differentiation of CD44^hi^ (hence effector) CD4^+^ T cells (Choi et al., 2005; Li et al., 2005), and forced expression of MHC-I leads to the development of PLZF^+^ CD8^+^ T cells (Georgiev et al., 2021). Thus, the innate-like features of MAIT cells are directly controlled by the expression of MR1 by thymic hematopoietic cells. It should be noted that in mice, MR1 is also expressed in cortical and medullary epithelial cells (Legoux et al., 2019a), enabling positive selection and differentiation of T cells expressing a MAIT TCR but harboring a naïve phenotype (Legoux et al., 2019b). Positive selection by other thymocytes is believed to enable homotypic engagement of signaling lymphocyte activating molecule (SLAM) family molecules, leading to signaling through the SLAM-associated protein (SAP) (Griewank et al., 2007). MAIT cells, like iNKT cells and like PLZF^+^ T cells in mice with ectopic expression of MHC-I by thymocytes, do not express PLZF in SAP-deficient mice (Georgiev et al., 2021; Koay et al., 2019; Legoux et al., 2019b; Nichols et al., 2005), suggesting that SLAM-SAP signaling drives PLZF expression. In support of this hypothesis, in vitro stimulation of immature thymocytes with an anti-Slamf6 antibody together with anti-CD3 can induce PLZF expression (Dutta et al., 2013; Legoux et al., 2019b; Tuttle et al., 2018). The molecular mechanisms controlling PLZF induction upon SLAM–SAP signaling remain elusive. Slamf6 triggering in vitro synergizes with CD3 triggering to yield high expression of the transcription factor Egr2, which binds to the PLZF promoter and induces PLZF expression upon ectopic expression in vitro (Seiler et al., 2012). However, SLAM family molecules can also inhibit TCR signaling in vivo (Lu et al., 2019), suggesting more complex regulation of Egr2 induction upon positive selection by DP thymocytes in vivo.

We recently used species-specific MR1 tetramers coupled to single-cell RNA sequencing to analyze MAIT cell development in five species other than mice (Bugaut et al., 2024). PLZF is induced in 5-OP-RU-specific thymocytes from all species, including the marsupial opossum which common ancestor with placental mammals that lived 110 MYA (Bi et al., 2018). Thus, T cells specific for 5-OP-RU have acquired innate-like features in the thymus since the emergence of mammalian therians (encompassing both placental and marsupial lineages) about 200 MYA (Álvarez-Carretero et al., 2022), i.e., immediately or rapidly after the emergence of the MR1 gene and the ability to present 5-OP-RU to the thymocytes. Among the other transcription factors identified as MAIT-specific and conserved across species is EZH2, which can methylate PLZF at position K430 leading to PLZF degradation in 293T cells (Vasanthakumar et al., 2017). *EZH2* is downregulated during MAIT cell maturation in all species, consistent with the stabilization of PLZF in mature MAIT cells. Despite these shared features, it is unclear whether PLZF induction relies on mechanisms that are conserved across species. In particular, PLZF-expressing MAIT cells are present at normal frequencies in the blood of SAP-deficient patients (Martin et al., 2009), indicating that PLZF can be induced through SAP-independent mechanisms in human MAIT cells.

In the opossum, human, cattle, and sheep, MAIT cell maturation in the thymus results in the acquisition of an effector program marked by expression of both *TBX21* (coding for Tbet) and *RORC* (coding for RORγt) (Bugaut et al., 2024). Human MAIT cells can exert effector functions as diverse as cytotoxicity and tissue repair but there are no discreet MAIT subsets in humans. Instead, MAIT cells form a homogeneous population that adapts to each tissue and context resulting in a variety of functional responses (Garner et al., 2023). By contrast, distinct type-1 and type-17 MAIT cell subsets develop in the rodent thymus (Koay et al., 2019; Legoux et al., 2019b; Rahimpour et al., 2015). The mechanisms driving MAIT1 versus MAIT17 lineage decisions in rodents are unclear but are likely independent of PLZF since *ZBTB16* expression is followed by *TBX21* and *RORC* co-expression in other species. MAIT1 and MAIT17 subsets from individual mouse thymi can display identical TCR clones, arguing against a TCR-instructive mechanism for the MAIT1 versus MAIT17 sublineage decision in rodents (Karnaukhov et al., 2024).

The development of MAIT cells, in particular the MAIT17 subset, is severely impaired in germ-free mice (Koay et al., 2016; Legoux et al., 2019a) and requires colonization with riboflavin-competent bacterial strains such as *E. coli*, *Enterococcus hirae*, or *Proteus mirabilis* (Constantinides et al., 2019; Legoux et al., 2019a). In *E. coli*, *ribD* is required for complete MAIT cell maturation while *ribE* is dispensable (Legoux et al., 2019a). Administration of 5-OP-RU, but not 5-A-RU or the MAIT antagonist acetyl-6-formylpterin (Ac6FP), to mice mono-colonized with Δ*ribD E. coli* restores MAIT cell development demonstrating a key role for 5-A-RU-derived agonist ligands in controlling MAIT cell maturation in the mouse thymus (Fig. 1). These results also suggest that mouse methylglyoxal does not react with 5-A-RU to form 5-OP-RU in vivo. In addition to 5-A-RU-derived ligands, MAIT cell development can be influenced by the self-derived ligand CA7S presented by MR1 (Ito et al., 2024). Although CA7S is a relatively weak MAIT ligand, it is very abundant and can reach 2 nmol/mg in the cecum (Chaudhari et al., 2021), suggesting a potential impact on MAIT cell biology. Indeed, MAIT cell development is impaired in the thymus of sulfotransferase knock-out mice, which lack CA7S (Ito et al., 2024). It should be noted that CA7S concentrations are considerably lower in the thymus (0.14 pmol/mg) as compared with the cecum, consistent with the inability of CA7S to cross the intestinal epithelium (Chaudhari et al., 2021).

A few mature MAIT cells able to produce IFNγ or IL-17 develop in the thymus of germ-free mice (Legoux et al., 2019a), demonstrating positive selection in the absence of 5-A-RU-derived ligands. MAIT1 and MAIT17 cells still develop in the thymus of germ-free mice that are deficient in sulfotransferases (Ito et al., 2024), ruling out a role for CA7S in their positive selection. Thus, the endogenous ligand mediating positive selection of MAIT cells in germ-free conditions remains to be identified. In humans, MAIT cells expressing PLZF are present in cord blood (Ben Youssef et al., 2018) indicating positive selection before birth. However, cord blood MAIT cells display a naïve phenotype and are very few, suggesting that human MAIT cells may be selected by CA7S or by other self-ligand(s) as in germ-free mice.

Upon thymic egress, MAIT cells preferentially home to non-lymphoid tissues such as skin, liver, lung, and gut lamina propria (Salou et al., 2018). In parabiosis experiments, about 20% of MAIT17 cells from liver and lung are of blood origin after a 5-wk period (Salou et al., 2018). In skin, these numbers are even higher with about 60% of MAIT cells originating from blood (du Halgouet et al., 2023). Thus, blood MAIT cells contribute significantly to the maintenance of tissue MAIT cells in mice. In humans, MAIT cells acquire a memory phenotype rapidly after birth and gradually increase in numbers over time to reach very high frequencies (1–4% of T cells in the blood) at 6–10 years of age (Ben Youssef et al., 2018). MAIT cell expansion is associated with a narrowing of the TCR repertoire (Ben Youssef et al., 2018) while the MAIT TCR β repertoire is somewhat specific to each individual (Garner et al., 2023), presumably resulting from the random clonal selection of few MAIT clonotypes in response to antigen stimulation during childhood. Within a given donor, MAIT cell TCR β repertoires are shared between blood and liver, arguing in favor of exchanges of cells between these two compartments in humans (Garner et al., 2023). Thus, MAIT cells in mice and humans probably circulate between blood and liver and possibly other non-lymphoid tissues such as skin.

Thus, MAIT cell accumulation over time in humans may result from repeated encounters with riboflavin-synthesizing pathogens or from low-grade “tonic” stimulation with microbiota-derived ligands. MAIT cells do not expand in laboratory mice, even after colonization with a human microbiota (Cui et al., 2015), arguing against a role for human specific microbiota-derived ligands in MAIT cell expansion over time. Alternatively, the self-derived ligand CA7S could contribute to shaping MAIT cell populations over extended periods of time. MAIT cells are undetectable in the blood of an adult patient with a homozygous R9H mutation in MR1 that abrogates 5-OP-RU binding, but not Ac6FP, suggesting that MAIT cell selection, expansion or survival in humans relies on 5-OP-RU or structurally similar ligands (Howson et al., 2020). It is unknown whether CA7S can bind to MR1^R9H^. Thus, MAIT cell ligands may play critical roles in establishing MAIT cell populations, acting either directly in the thymus and/or in peripheral tissues, yet the nature and the origin (self or microbial derived) of these ligands remains elusive. The role of MAIT ligands in MAIT cell homing to and from tissues also deserves further exploration.

## TCR-dependent functions of MAIT cells

MAIT cells constitutively express cytokine receptors enabling responses to IL-12, IL-15, IL-18, IL-23, type I interferons or IFNγ and thus can be activated in a TCR-independent manner. This mode of activation enables MAIT cell contribution to protection against pathogens lacking MAIT ligand production such as viruses (Provine et al., 2021; Van Wilgenburg et al., 2018; van Wilgenburg et al., 2016) but also against *Klebsiella pneumoniae* which possess a riboflavin biosynthesis pathway (López-Rodríguez et al., 2023). Here we will focus on MAIT cell functions that are TCR-dependent, with the idea that these functions are more likely to be responsible for MAIT cell conservation across species.

### Role of MAIT cells in bacterial infections

The high frequencies of MAIT cells in barrier tissues, together with their ability to react immediately to antigenic stimulation, makes them well positioned as first responders against bacterial infections. MAIT cell blood frequencies decrease in patients with pulmonary bacterial infections and MAIT cells can be detected in sites of infection, suggesting recruitment and potential involvement in antibacterial responses (Le Bourhis et al., 2010). In vitro, TCR stimulation of human MAIT cells in the presence of bacteria drives proliferation (Kurioka et al., 2015) and contributes (in synergy with non-cognate signals) to rapid FasL expression and IFNγ secretion (Lamichhane et al., 2019). Degranulation of human MAIT cells in response to *E. coli* relies on TCR stimulation alone and cannot be blocked by anti-IL-12 or IL-18 antibodies (Kurioka et al., 2015). Accordingly, human MAIT cells can kill *E. coli* and *Shigella flexneri*–infected target cells in an MR1-dependent manner (Boulouis et al., 2020; Kurioka et al., 2015; Le Bourhis et al., 2013). MAIT cells can also directly kill free *E. coli* through the release of granulysin (Boulouis et al., 2020). Granulysin is not conserved in rodents and it is unclear whether mouse MAIT cells can kill free bacteria.

In vivo, pulmonary infection with riboflavin-competent *Salmonella enterica* Typhimurium induces robust proliferation of MAIT cells, which can make up to 50% of lung αβ T cells (Chen et al., 2017). Rib-deficient *Salmonella* fail to induce MAIT cell expansion, demonstrating a role for TCR ligands (Chen et al., 2017). Accordingly, intranasal instillation of synthetic 5-OP-RU in combination with TLR ligands or with IL-23 drives pulmonary MAIT cell expansion while 5-OP-RU or TLR ligands alone do not (Chen et al., 2017; Wang et al., 2018, 2019). The other rib-competent pathogens *Francisela tularensis* and *Legionella longbeachae* also induce MAIT cell expansion upon infection (Meierovics et al., 2013; Wang et al., 2018; Zhao et al., 2021).

*Mr1*^−/−^ mice, lacking MAIT cells, are more susceptible to pulmonary or systemic infection with *F*. *tularensis* (Meierovics et al., 2013; Zhao et al., 2021), *L. longbeachae* (Wang et al., 2019), *E. coli* and *Mycobacterium abscessus* (Le Bourhis et al., 2010). *S*. Typhimurium bacterial load is not better controlled in WT mice than in *Mr1*^−/−^ mice despite robust MAIT cell expansion in lungs of WT mice (Chen et al., 2017). The role of cognate recognition in the antimicrobial properties of MAIT cells in vivo is not completely clear. Upon MAIT cell adoptive transfer into *RAG2*^−/−^γc^−/−^ recipient mice and subsequent infection with *L. longbeachea,* anti-MR1 antibodies reduce the protective antimicrobial effect of MAIT cells (Wang et al., 2018), suggesting a role for cognate recognition. In the same setting, MAIT-intrinsic IL-17, perforin, granzymes, and TNF are dispensable for protection while IFNγ is required (Wang et al., 2018). In *F. tularensis* infection, MAIT cells adoptively transferred in *RAG2*^−/−^γc^−/−^ recipients mediate antimicrobial protection dependent on intrinsic IFNγ, TNF, and GM-CSF but not IL-17 (Zhao et al., 2021). Whether MAIT ligands are required for protection against the *F. tularensis* live vaccine strain is unknown. Reduced synthesis of MAIT ligands in the *F. tularensis novicida* subspecies leads to reduced MAIT cell expansion upon pulmonary infection, higher bacterial loads and increased mortality (Shibata et al., 2022). Altogether, TCR stimulation of MAIT cells induces robust expansion in infected tissues and can contribute to bacterial pathogen control. MAIT cells produce IFNγ, TNF, and GM-CSF upon bacterial infections suggesting a role in the recruitment of antimicrobial innate immune cells such as macrophages and neutrophils.

In chronic infections, the proinflammatory properties of MAIT cells can have deleterious effects, as evidenced in a chronic *H*.* pylori* infection model (D’Souza et al., 2018), in which long-term infection leads to stomach atrophy. In that model, *Mr1*^+^ mice suffer worst gastritis than *Mr1*^−/−^ counterparts, particularly when MAIT cells are first expanded by intranasal infection with *S.* Typhimurium prior to *H. pylori* infection. While MAIT ligands are clearly required for MAIT cell expansion, the role of TCR signals in sustaining chronic gastric inflammation is unknown.

Since riboflavin is ubiquitously present in animal hosts, bacterial pathogens able to import riboflavin and harboring a riboswitch could shut down MAIT ligand synthesis in vivo. Other mechanisms, or variations in riboswitch efficiency, could lead to variations in the synthesis of riboflavin and MAIT ligands by individual bacteria. For example, clinical isolates of *Streptococcus pneumoniae* produce various amounts of riboflavin which correlate with the amounts of MAIT ligands produced in vitro (Hartmann et al., 2018). Similarly, an isolate from the invasive *S. *Typhimurium pathovar 313 over-expresses *ribB* as compared with the pathovar 19, correlating with reduced MAIT ligand synthesis (Preciado-Llanes et al., 2020). Over-expression of *ribB* in *S. *Typhimurium 19 is sufficient to reduce MAIT ligand synthesis and MAIT cell activation, presumably by increasing consumption of 5-A-RU to produce riboflavin, instead of 5-OP/OE-RU (Preciado-Llanes et al., 2020). Lastly, while a free-living strain of *F. tularensis* produces MAIT ligands, the pathogenic *subsp. tularensis* does not despite normal expression of all rib genes (Shibata et al., 2022). Five amino-acid substitutions in the *ribD* enzyme from the pathogenic strain are sufficient to reduce MAIT ligand synthesis when introduced in the free-living strain (Shibata et al., 2022) suggesting sequence variations that affect *ribD* function. Of note, *ribD* is dispensable for the growth of *F. tularensis subsp. novicida* in vitro. Mice infected with the free-living strain expressing this poorly functional *ribD* succumb to infection more rapidly than mice infected with WT free-living *F. tularensis,* suggesting increased pathogenesis conferred by the lack of MAIT ligand synthesis (Shibata et al., 2022).

Altogether, pathogen-derived MAIT ligands could contribute to the antibacterial functions of MAIT cells by boosting local MAIT cell numbers and increasing the magnitude and speed of the response. MAIT cell recognition of pathogen-derived ligands may constitute a selection pressure on the pathogens driving the emergence of variants with reduced or abrogated synthesis of riboflavin pathway-derived ligands. However, it remains unclear whether MAIT cells are evolutionarily conserved because of their antibacterial functions, given the relatively late emergence of MAIT cells in evolution and their loss in several mammalian groups (Boudinot et al., 2016). Besides pathogens, MAIT ligands can be abundantly produced by commensal bacteria (El Morr et al., 2024; Tastan et al., 2018), pointing to a potential role in the maintenance of mucosal tissue integrity.

### Role of MAIT cells at the skin barrier

Human MAIT cells stimulated in vitro with 5-OP-RU, but also mouse MAIT cells in response to pulmonary *L. longbeachae* infection, express genes associated with tissue-repair functions (Hinks et al., 2019; Leng et al., 2019). Specifically, MAIT cell cognate stimulation drives expression of a gene signature defined in commensal-specific skin Th17 cells (Linehan et al., 2018) and associated with angiogenesis, tissue remodeling and wound healing. Expression of this tissue-repair gene signature is not induced directly in the thymus, but is acquired at steady-state in MAIT cells from the skin, mesenteric lymph nodes and intestine (Bugaut et al., 2024; du Halgouet et al., 2023). Mouse colonization with the skin commensal *Staphylococcus epidermidis* further drives expression of tissue-repair genes in skin MAIT cells (Constantinides et al., 2019). Whether MAIT cells from other tissues acquire this signature at steady-state is unclear. MAIT cells from the skin and intestine express high levels of the TCR signaling marker *Nr4a1* (coding for Nur77) at steady-state (du Halgouet et al., 2023; El Morr et al., 2024), presumably due to local production of TCR ligands by the microbiota, which may control expression of the tissue-repair gene set.

In the mouse skin, MAIT cells are present in the dermis at frequencies that vary according to the composition of the skin microbiota (Constantinides et al., 2019). Skin colonization with *S*.* epidermidis* results in MAIT cell expansion, even in LTα-deficient mice, which lack lymph nodes, suggesting local proliferation in the skin. Deletion of either *S. epidermidis ribD* or *Mr1* abrogates MAIT cell expansion demonstrating the key role of MAIT ligands in tuning MAIT cell abundance in the skin. In a skin wound healing model, skin MAIT cells do not over-express tissue-repair genes, but expand during skin injury (du Halgouet et al., 2023). Re-epithelialization and wound closure occur more slowly in *Mr1*^−/−^ mice than in *Mr1*^+^ counterparts (Constantinides et al., 2019; du Halgouet et al., 2023). Skin injury induces production of the epidermal growth factor amphiregulin (Areg) by MAIT cells, and Areg deletion in MAIT cells delays skin wound closure, pointing to Areg secretion as an important mediator of MAIT cell tissue-repair functions in the skin (du Halgouet et al., 2023). MAIT cells adoptively transferred into *Cd3e*^−/−^ recipients accelerate wound healing independently of *Mr1* expression by the host (du Halgouet et al., 2023). Thus, while TCR signals in MAIT cells may be required to acquire a tissue-repair program, this program may be expressed and function independently of MR1.

In addition to direct effects on epithelial cells, MAIT cells could promote tissue integrity through the recruitment of other effector cells. For example, TCR or cytokine-stimulated human MAIT cells release neutrophil-attracting chemokines (Lamichhane et al., 2019). In a mouse model of atopic dermatitis, cognate MAIT cell activation leads to the activation of eosinophils and local recruitment of IL-4 and IL-13-producing cells (Naidoo et al., 2021). Thus, MAIT cell TCR stimulation can contribute to immune cell chemotaxis. It is unclear whether microbiota shifts associated with atopic dermatitis result in altered synthesis of MAIT ligands.

### Role of MAIT cells at the intestinal barrier

MAIT cells can represent up to 40% of T cells in the human liver (Dusseaux et al., 2011; Kammann et al., 2024). In the mouse liver, MAIT cells are less abundant and present a mixed phenotype of type 1, type 17, and blended 1/17 (Bugaut et al., 2024; Salou et al., 2018). Their exact location in the liver (parenchyma or intravascular) has not been determined. In mice, it is likely that MAIT1 cells are in the intravascular space like NKT1 cells (Geissmann et al., 2005) while MAIT17 could be in the parenchyma. Notably, biliary epithelial cells can present bacterial antigens to MAIT cells (Jeffery et al., 2016). In both species, given their antibacterial specificity and specific location in the sinusoid, MAIT and in particular MAIT1 cells could play the role of firewall against bacteria coming from the intestine to the liver through portal blood. Moreover, MAIT cells may be involved in various physio-pathological processes such as fibrosis (Hegde et al., 2018; Mabire et al., 2023) through inadequate activation of the repair program of MAIT17 cells and spontaneous or therapeutically induced antitumor defense (Ruf et al., 2021) or antiviral infection (Ussher et al., 2018).

MAIT cells are found in the small and large intestine lamina propria, where they all express RORγt (Rouxel et al., 2017; Salou et al., 2018). In addition to RORγt, MAIT cells can express Tbet and acquire a blended type 1 and type 17 transcriptional program associated with the expression of tissue repair and cytotoxicity genes (Bugaut et al., 2024), which suggests versatile functions in the gut depending on the context. Thymic MAIT17, but not MAIT1 cells, can give rise to MAIT1/17 cells in the gut lamina propria upon adoptive transfer into *Rag2*^*−/−*^ recipients (Bugaut et al., 2024), suggesting they originate from thymic MAIT17 cells. The mechanisms controlling the maturation of MAIT17 cells in the gut remain unclear and could involve IL-23 and MyD88-dependent signals.

On the NOD background, *Mr1*^−/−^ mice present greater intestinal permeability than their *Mr1*^+^ counterparts, as evidenced by higher FITC-dextran concentrations found in the blood after oral gavage (Rouxel et al., 2017). The lack of MAIT cells is also associated with lower expression of the genes coding for occludin and Muc2 in the ileum, suggesting a key role for MAIT cells in the maintenance of gut barrier integrity in the NOD mouse. MAIT cells seem to play a similar role in the B6 background since *Mr1*^+^ mice express higher levels of the tight junction protein-encoding genes *Cldn4* and *Cldn8* in the colon as compared with *Mr1*^−/−^ mice (Varelias et al., 2018). In an acute graft-versus-host disease model, the intestinal barrier is more permeable to FITC-dextran and the colitis is more severe in the absence of recipient MAIT cells (Varelias et al., 2018), suggesting that MAIT cells contribute to the maintenance of the intestinal barrier also on a B6 background. Similarly in a xenogeneic graft versus host disease (GVHD) model, human MAIT cells potently inhibit alloreactive T cell proliferation and activation in a TCR-dependent manner (Talvard-Balland et al., 2024).

**Figure 2.**
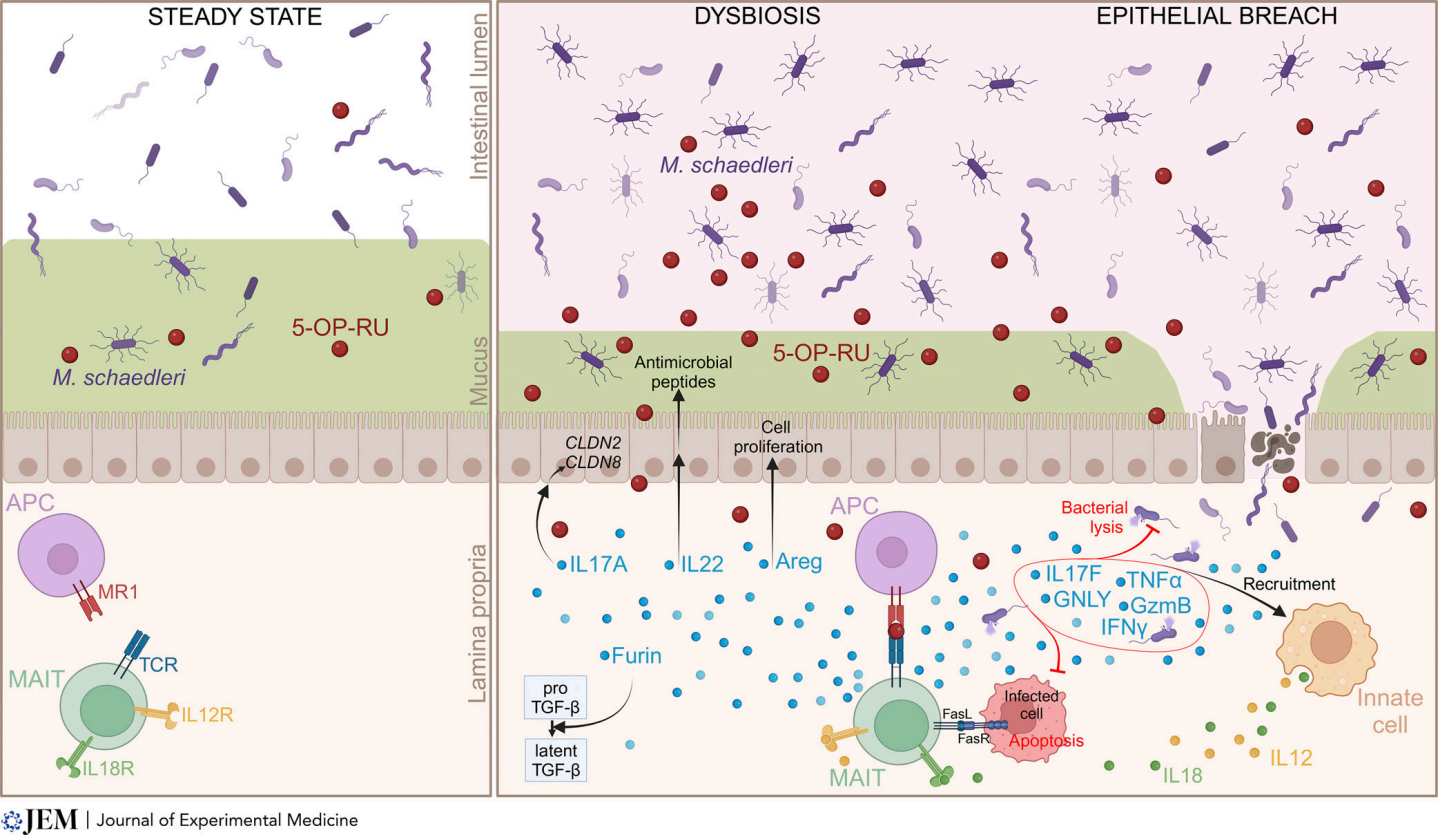
**Barrier-promoting and antimicrobial functions of MAIT cells at the intestinal barrier.** Intestinal dysbiosis leads to an expansion of ribD-expressing bacteria, particularly *M. schaedleri*, resulting in the synthesis of MAIT ligands and the activation of MAIT cells in the lamina propria. During colitis, MAIT cells produce barrier-promoting factors such as IL17A, a cytokine that induces the expression of tight junction proteins (claudin 8 and 2) in epithelial cells; amphiregulin, which stimulates epithelial cell proliferation through the epidermal growth factor receptor; and Furin, involved in TGFβ maturation. MAIT cells also provide antimicrobial mediators, such as IL22, which stimulates epithelial production of antimicrobial peptides, and the inflammatory cytokines IL17F, TNFα, granulysine, granzyme B, and IFNγ, which promote the recruitment of innate immune cells and contribute to bacterial lysis. MAIT cells can also express FasL, leading to FasR-mediated apoptosis of infected cells. Created with Biorender.

In a chronic model of chemically induced colitis, in which mice are subjected to repeated cycles of colitis induced by DSS to mimic the inflammatory flare typically observed in human inflammatory bowel diseases (IBD), *Mr1*^−/−^ mice lose more weight and suffer worse epithelial damage than their *Mr1*^+^ cage mates (El Morr et al., 2024), indicating that the presence of MAIT cells is associated with reduced colitis severity in this setting. In the azoxymethane (AOM)/DSS model of colitis-induced colorectal cancer, rectal tumor development is enhanced in mice lacking MAIT cells as compared with *Mr1*^+^ cage mates, further emphasizing the anti-inflammatory role of MAIT cells upon DSS treatment (El Morr et al., 2024). Of note, the growth of the colon tumor cell line MC38 is similar in *Mr1*^+^ and *Mr1*^−/−^ mice upon intrarectal transplantation, arguing against a direct antitumor effect of MAIT cells in the colon. A subset of MAIT cells presents a unique transcriptional profile in the colon of mice with chronic DSS-induced colitis, marked by the overexpression of genes associated with cytotoxicity (*Gzmb*, *Srgn*), chemotaxis (*Cxcl2*), barrier promotion (*Il22*, *Il17a*) and wound healing (*Furin*, *Hif1a*, *Ctla4*) (Fig. 2). Colonic MAIT cells also express *Areg* upon chronic colitis (El Morr et al., 2024). The effector mechanisms mediating MAIT cell protective effect against chronic colitis remain to be determined and could involve several of these molecules.

In agreement with the notion that MAIT cells could exert versatile functions in the gut, MAIT cells can be associated with reduced epithelial barrier integrity in some models. In B6 mice fed on a high-fat diet, the lack of MAIT cells is associated with reduced intestinal permeability (estimated again by FITC-dextran dosage in serum after oral gavage) and greater expression of *Cldn4*, *Ocln*, and *Muc2* in ileum epithelial cells, suggesting that MAIT cells reduce intestinal permeability in this setting (Toubal et al., 2020). In the acute model of ulcerative colitis induced by oxazolone, which relies on haptenization of colonic antigens and induces rapid weight loss, *Mr1*^−/−^ mice present reduced histopathology, reduced weight loss, and improved survival as compared with controls (Yasutomi et al., 2022), suggesting a deleterious effect of MAIT cells in this model.

In healthy human donors, MAIT cells isolated from the ileum, cecum, and colon present a distinct phenotype characterized by expression of the ecto-enzyme CD39, which promotes an immunosuppressive environment by degrading extracellular ATP (Kammann et al., 2024). Human intestinal MAIT cells secrete IL-22 spontaneously and secrete IL-10 and IL-17A upon PMA-ionomycin stimulation, consistent with barrier-promoting functions (Kammann et al., 2024). In two independent studies of patients with Crohn’s disease or ulcerative colitis, MAIT cell frequencies are decreased in the blood but increased in inflamed intestinal lesions, suggesting recruitment (Haga et al., 2016; Serriari et al., 2014). MAIT cells from the blood of IBD patients over-produce TNFα in one study (Tominaga et al., 2017) and IL-22 in another study (Serriari et al., 2014). All studies of MAIT cells in IBD reported increased secretion of IL-17 by blood MAIT cells during IBD (Haga et al., 2016; Serriari et al., 2014; Tominaga et al., 2017). IL-17 blockade exacerbates disease in IBD patients, suggesting a protective function (Fauny et al., 2020).

Altogether, MAIT cells appear protective during colitis but may exert proinflammatory functions in other contexts such as during obesity. Further work should attempt to identify the factors controlling the divergent effector functions of MAIT cells in each context.

## Conclusions

The reasons for the evolutionary conservation of MAIT cells remain unknown in the absence of a known pathology linked to a genetic deficiency of the MAIT/MR1 axis in humans. Given their antibacterial capacities, which may be more evident in humans than in mice, MAIT cells may have provided a selective advantage against pathogenic bacteria relying on strong riboflavin production for their pathogenicity. These pathogens may have disappeared in our current environment. Alternatively, the unusual properties of MAIT ligands, which can cross intact epithelia to activate underlying MAIT cells, may provide a unique way for T cells to sense the rapidly evolving ecology of the gut lumen prior to any breach in the integrity of the gut barrier. The ensuing response may stimulate the secretion of anti-microbial peptides, mucus proteins, and tight junction components in anticipation of a potential barrier leakage. These two possibilities are not mutually exclusive, and given the versatile functions of MAIT cells in the gut, MAIT cell cytotoxicity may become critical to contain commensal bacteria upon rupture of the intestinal epithelium.

Despite the rapidly increasing knowledge of the biology of MAIT cells, key questions remain unsolved, in particular regarding the identity and function of self-ligands, the regulation of ligand distribution in the body, and mechanisms of transport in vivo. In addition, understanding how the various and seemingly antagonistic functions of MAIT cells are orchestrated in a given tissue will be key to harnessing the potential of these cells for therapeutic purposes.
